# Apigenin-7-*O*-β-d-(6″-*p*-coumaroyl)-glucopyranoside reduces myocardial ischaemia/reperfusion injury in an experimental model via regulating the inflammation response

**DOI:** 10.1080/13880209.2019.1701043

**Published:** 2019-12-30

**Authors:** Wei Quan, Shanbo Ma, Yanrong Zhu, Qing Shao, Jixing Hou, Xiaoqiang Li

**Affiliations:** a Key Laboratory of Gastrointestinal Pharmacology of Chinese Materia Medica of the State Administration of Traditional Chinese Medicine, Department of Pharmacology, School of Pharmacy, Air Force Medical University, Xi’an, China; b Xi’an Mental Health Center, School of Medicine, Xi’an Jiaotong University, Xi’an, China; c Department of Pharmacy, Xijing Hospital, Air Force Medical University, Xi’an, China

**Keywords:** Flavonoid glycosides, NF-κB, cardioprotection, APG

## Abstract

**Context:**

Traditionally, *Clematis tangutica* Korsh. (Ranunculaceae) is used as a Tibetan herb for treating indigestion and blood stasis in China. Recently, a flavonoid glycoside, apigenin-7-*O*-β-d-(6″-*p*-coumaroyl)-glucopyranoside (APG), was isolated from the whole plant of *C. tangutica*.

**Objective:**

To investigate the cardioprotective effects of APG against myocardial ischaemia/reperfusion injury (MI/RI) and the possible mechanism.

**Materials and methods:**

Animals were subjected to 30 min/3 h MI/RI model. At the end of reperfusion, infarct size (IS), histopathology, serum levels CK-MB, LDH, TNF-α, IL-6 and MPO activities were detected. Phospho-IκB-α, ICAM-1 and NF-κB were assessed *in vivo*. Neonatal rat cardiomyocytes were pre-treated with or without APG, followed by stimulation with 8 h/2 h oxygen and glucose deprived/reoxygenation (OGD/R) model. Cell viability, LDH and cardiomyocyte apoptosis were assessed. The expression levels of phospho-IκB-α and NF-κB were measured *in vitro*.

**Results:**

Treatment with APG significantly reduced the following indicators *in vivo* (*p* < 0.05): (1) the IS (16.2%); (2) morphology score (1.67); (3) myocardial injury enzymes: CK-MB (26.2 ng/mL) and LDH (688 U/L); (4) pro-inflammatory cytokines: TNF-α (31.5 pg/mL) and IL-6 (163.8 pg/mL); (5) MPO activity (2.75 U/mg); (6) expression levels of phospho-IκB-α (0.47), NF-κB (2.87) and ICAM-1 (10.2). Moreover, treatment with APG also remarkably (*p* < 0.05) attenuated the following indicators *in vitro*: (1) LDH level (206 U/L); (2) cardiomyocyte apoptosis; (3) phospho-IκB-α (1.37) and NF-κB (2.50).

**Conclusions:**

APG possesses protective effects against MI/RI injury in rats and OGD/R-induced injury in cardiomyocytes by suppressing translocation of NF-κB and reducing inflammatory response; consequently, APG is helpful for treatment of ischaemic heart disease.

## Introduction

Ischaemic heart disease (IHD) secondary to acute myocardial infarction is one of the leading causes of morbidity and mortality worldwide. Percutaneous coronary intervention, angioplasty and fibrinolytic therapy can effectively reduce the early mortality associated with acute myocardial infarction. However, myocardial ischaemia/reperfusion injury (MI/RI) is frequently observed in clinical practice, which is a major cause of further heart events in clinic. MI/RI is a process that produces irreversible injuries (reperfusion) from reversible injuries (ischaemia). Studies conducted on animal models of acute myocardial infarction demonstrated that lethal reperfusion injury accounts for up to 50% of the final size of a myocardial infarct (Yellon and Hausenloy 2007). Thus, effective therapeutic strategies targeting MI/RI is urgently required to improve the prognosis of IHD.

**Figure 1.**
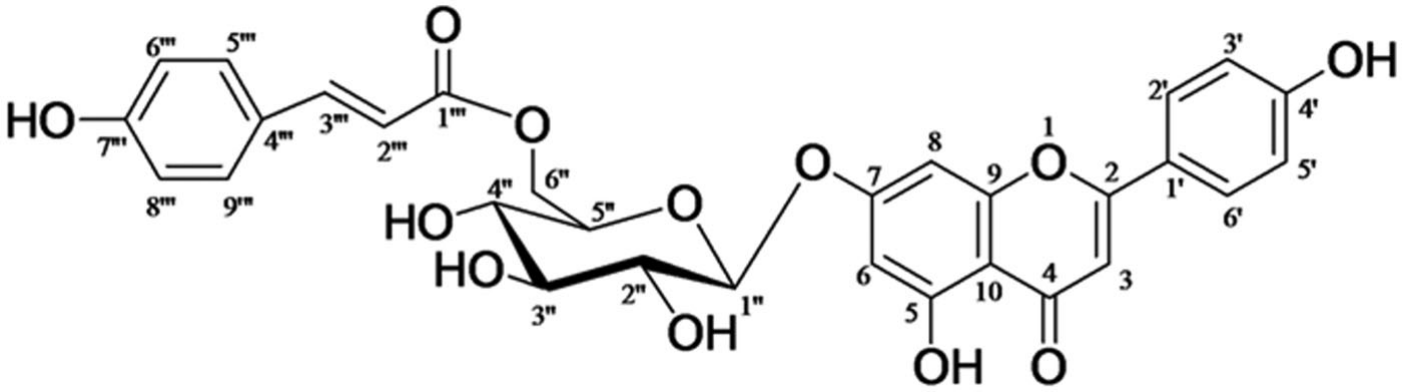
Chemical structure of apigenin-7-*O*-β-d-(6″-*p*-coumaroyl)-glucopyranoside.

In recent years, natural products derived from plants originating from unusual environments, such as high-altitude area in Tibet, have markedly aroused scholars’ attention. *Clematis tangutica* Korsh. (Ranunculaceae) has been widespread in the southwest mountain area in China. As reported, the species of the genus *Clematis* emerged as significant source of traditional medicines for the treatment of various ailments, and they have a wide range of constituents, such as triterpenes, flavonoids, lignans, etc. (Chawla et al. 2012). Traditionally, *Clematis tangutica* has been used for treating indigestion and blood stasis in Tibet (Yang 1991). It is the principal component in a Chinese patent medicine named ‘Kangtai’ capsule, which was extensively used for the treatment of cardiovascular and cerebrovascular diseases in China. Previously, phytochemical investigation of the whole plants of *Clematis tangutica* led to isolation of some new bioactive triterpenoid saponins in our laboratory (Zhang et al. 2013). Recently, a flavonoid glycoside, namely apigenin-7-*O*-β-d-(6″-*p*-coumaroyl)-glucopyranoside (APG) (Figure 1), was isolated from the whole plant of *C. tangutica*. To our knowledge, flavonoids have long been known to possess various pharmacological activities relevant to MI/RI, involving anti-inflammation, antioxidant, antiplatelet aggregation and vasorelaxation properties (Akhlaghi and Bandy 2009). Numerous studies have indicated that flavonoids are beneficial for reducing the damage of MI/RI both *in vivo* and *in vitro* (Amorini et al. 2003; Aneja et al. 2004; Fantinelli et al. 2005; Hotta et al. 2006; Hirai et al. 2007; Dong et al. 2011). A previous research found that APG could protect the brain from MI/RI, which was accompanied by reducing the infract size and ameliorating the neurological deficit in the rat middle cerebral artery occlusion model (Tang et al. 2014). Hence, we evaluated the protection of APG in a MI/RI model.

To our knowledge, MI/RI is associated with an inflammatory cascade that perpetuates further damage to cardiac tissue after ischaemia. It is widely accepted that the up-regulated transcription factor nuclear factor-kappa B (NF-κB) plays a pivotal role during inflammation process (Zhong et al. 2004). Thus, in the present study, we attempted to investigate whether APG can inhibit the inflammation and reduce the myocardium injury in both MI/RI rat model and oxygen-glucose deprivation/reoxygenation (OGD/R)-induced neonatal rat cardiomyocytes injury model.

## Materials and methods

### Ethics and surgical preparation of animals

Experimental protocols were approved by the Institutional Animal Care and Use Committee of the Air Force Medical University and were performed according to the National Institutes of Health Guide for the Care and Use of Laboratory Animals (NIH publication no. 85-23, National Institutes of Health Publication, Washington, DC, revised 1996). All efforts were made to minimize the number of the animals used and their suffering. All experiments were performed in a blinded, randomized manner.

Male Sprague-Dawley rats (body weight, 250–300 g; certificate no. SCKX 2007-007) were purchased from the Experimental Animal Research Center of Air Force Medical University. The rats were raised in a 12 h light–dark cycle and temperature-controlled room, and were all fasted (except for water) for 12 h prior to the experiment. Animals were anaesthetized by intraperitoneal injection of 60 mg/kg pentobarbital sodium. A left thoracotomy was performed then a 6/0 silk suture threaded through a vinyl tube was placed around the left anterior descending coronary artery (LAD) to form a reversible occlusion snare. Following occlusion for 30 min, reperfusion for 3 h was carried out by releasing the snare. Successful occlusion was confirmed by ST-segment elevation on electrocardiograms. Sham-operated rats underwent the same surgical procedures, without ligation of the 6/0 silk suture.

### Preparation and structural verification of APG

Whole *Clematis tangutica* plants were collected from Huangnan Tibetan Autonomous Prefecture in the southeast of Qinghai Province, China, at an altitude of approximately 3500 m above sea level. A voucher specimen was deposited in the Herbarium of the Department of Pharmacy, Xijing Hospital, Air Force Medical University, Xi’an, China. Air-dried powders obtained from whole *Clematis tangutica* plants (2 kg) were extracted with 3 L 70% EtOH under reflux for 2 h, and repeated three times (3 L × 3). Filtrates were combined and evaporated to dryness under vacuum. The residue was suspended in H_2_O and then partitioned successively with petroleum ether (4 L × 3) and *n*-BuOH (4 L × 4). The *n*-BuOH extract was subjected to column chromatography on silica gel (800 g, 15 × 60 cm), and was eluted with a CHCl_3_–MeOH–H_2_O gradient (from 20:1:0.1 to 6:3:0.3, lower phase) to give six crude fractions based on thin layer chromatography analysis. The fraction containing APG was concentrated under reduced pressure at 60 °C to yield crude extracts, and then, was further purified on a silica gel column (200 g, 3 × 80 cm) eluted with a CHCl_3_–MeOH–H_2_O gradient (from 7:3:1 to 6.5:3.5:1, lower phase) to obtain pure APG (content >99.1%). The structure of APG was verified by comparing nuclear magnetic resonance (NMR) data with those reported in the literature (Hideji et al. 1981; Delazar et al. 2005).

### Other materials

The anti-NF-κB (P65) antibody and anti-phospho-IκB antibody were purchased from Cell Signaling Technology (Beverly, MA). The anti-PCNA antibody and anti-beta actin antibody were purchased from Abcam (Cambridge, UK). The goat anti-rabbit and goat anti-mouse secondary antibodies were purchased from Zhongshan (Beijing, China). The Bio-Rad imaging system for western blots was purchased from Bio-Rad (Hercules, CA). Kits for measuring creatine kinase MB (CK-MB), lactate dehydrogenase (LDH), tumour necrosis factor-alpha (TNF-α) and interleukin-6 (IL-6) were purchased from the Institute of Jiancheng Bioengineering (Nanjing, China). The tissue myeloperoxidase (MPO) kit was purchased from the Institute of Jiancheng Bioengineering (Nanjing, China). TUNEL kits were purchased from Roche Molecular Biochemicals (Mannheim, Germany). Evans blue and triphenyl tetrazolium chloride (TTC) were purchased from Sigma-Aldrich (St. Louis, MO). Haematoxylin–eosin (H&E) was purchased from Solarbio (Beijing, China). The siRNAs for NF-κB (sense: GGACCUAUGAGACCUUCAATT, antisense: UUGAAGGUCUCAUAGGUCCTT) were designed and synthesized by Hippobio (Zhejiang, China). Lipofectamine 2000 transfection reagent was purchased from Invitrogen (Carlsbad, CA). pcDNA3.1(+)-NF-κB for overexpression of NF-κB was designed and synthesized by Fenghui Biology (Hunan, China).

### Experimental protocol in rat

In the present study, 60 rats were randomly assigned to five groups in equal proportions (*n* = 12 for each group), involving sham-operated, MI/RI + vehicle, MI/RI + APG (25, 50 and 100 mg/kg). Rats in sham-operated and MI/RI + vehicle groups received 0.2 mL/100 g volume of vehicle (5% DMSO, i.p.), and rats in MI/RI + APG groups received APG equal to 25, 50 and 100 mg/kg of body weight, respectively. All groups received the same volume of solutions by intraperitoneal injection for 10 min before the reperfusion course. The body temperature of rat was maintained at 37 °C using a heating pad during the operation. At the end of reperfusion, each group of rats was divided into two subgroups (*n* = 6 for each subgroup), in which one subgroup measured infarct size (IS) and other subgroup was used for determination of serum biochemical indices, histopathological analysis and western blot analysis.

### Determination of area at risk and infarct size

At the end of reperfusion, the left coronary artery was occluded again, and 2 mL of Evans blue dye (3% in saline) was injected into the jugular vein to outline the area at risk (AAR). The heart was rapidly excised and removed from the major vessels. Hearts were rinsed with cold saline, frozen at –20 °C, and then, cross-sectioned into six slices. Each slice was counterstained with 2% TTC for 10 min at 37 °C and fixed in 4% paraformaldehyde overnight. Afterwards, TTC stains viable tissue dark red, while the infarct portion remained greyish-white. The area of infarction was determined by Image-Pro^®^ Plus 6.0 software (Media Cybernetics, Bethesda, MD). The AAR was estimated as a percentage of the total left ventricle (LV). Similarly, the IS was estimated by the area as a percentage of the AAR.

### Histopathological examination

AAR tissue sections were fixed in 4% paraformaldehyde, then embedded into paraffin, and cut into 5 μm thick sections for histopathological analysis. Paraffin-embedded sections were stained with H&E, and morphological evaluation was undertaken by light microscopy. The extent of myocardial tissue injury was assessed in five random fields (×400 magnification). Histopathological damage was quantified by the following morphological criteria: score 0, no damage; score 1, focal areas of oedema and necrosis; score 2, patchy areas of myocardial cell swelling, necrosis and neutrophil infiltration; score 3, confluent areas of necrosis with the presence of contraction bands, neutrophil infiltration and compressed capillaries; and score 4, massive areas of necrosis, neutrophil infiltration, compressed capillaries and haemorrhage (Zingarelli et al. 1998). The histopathological examination and evaluation were performed by a pathologist who was blind to our experimental protocol.

### Determination of serum levels of CK-MB, LDH, TNF-α and IL-6

Blood samples were collected at the end of reperfusion via abdominal aorta and then centrifuged at 4000×*g* for 15 min at 4 °C to obtain serum (TDZ4A-WS, Xiangyi, China). Serum levels of CK-MB, LDH, TNF-α and IL-6 were measured by an enzyme-linked immunosorbent assay (ELISA) kit according to the manufacturer’s instructions. Besides, CK-MB was expressed as ng/mL, LDH was expressed as U/L, and IL-6 and TNF-α were expressed as pg/mL.

### Determination of MPO activity

Myocardial tissues from the infarct zone were saved. The tissues were homogenized in 5.0 mL of 0.1 M Tris–HCl buffer (pH = 7.4) in ice-cold conditions. The activity of MPO in tissue was measured using an ELISA kit according to the manufacturer’s instructions. MPO was expressed as U/mg.

### Cell culture and simulated ischaemia/reperfusion injury model

Neonatal cardiomyocytes were obtained from 1- to 2-day-old Sprague-Dawley rats. Cells were maintained in Dulbecco’s modified Eagle’s medium (DMEM, Gibco, New York, NY) with 10% foetal bovine serum (FBS; Gibco, New York, NY), 100 U/mL penicillin, 100 μg/mL streptomycin and cultured at 37 °C in a 5% CO_2_ incubator for 72 h. Cells were then pre-treated with or without APG (2, 4 and 6 μM, solved in 0.5% DMSO) for another 3 h. The OGD technique was applied based on a previously described protocol (Yan et al. 2017). In the present study, the OGD injury was produced by incubating with blank solution and exposed to a hypoxic environment of 95% N_2_ and 5% CO_2_ in airtight gas chambers at 37 °C for 8 h (Billups-Rothenberg, Los Angeles, CA). After OGD treatment, cells were removed from the gas chambers, and the OGD solution was replaced with warmed culture medium for 2 h (recovery period) in a CO_2_ incubator at 37 °C.

### Cell transfection

The cardiomyocytes were cultured in DMEM supplemented with 10% FBS, streptomycin (100 μg/mL) and penicillin (100 U/mL) at 37 °C and 5% CO_2_. For transfection, cardiomyocytes (5 × 10^4^) were seeded in six-well plates. Then, the cells were transfected with siRNA-NF-κB and pcDNA3.1(+)-NF-κB using the Lipofectamine 2000 transfection reagent for 6 h in medium lacking antibiotics, and the cells were washed in a warmed medium and maintained at least 48 h for the next experiments.

### Analysis of cell viability

Cell viability was detected by microculture tetrazolium (MTT) assay. The cardiomyocytes were seeded at a density of 4 × 10^4^ cells/well in 96-well plates. After different treatments, 20 μL of the MTT solution (5 mg/mL) was added into each well and the final concentration of 5 mg/mL was kept for 2 h at 37 °C. After that, the medium was removed and DMSO (150 mL) was added into each well. The optical density (OD) was recorded spectrophotometrically at 490 nm with a microplate reader (Infinite M200 PRO, Männedorf, Switzerland). Cell viability was expressed as a percentage.

### Determination of LDH release in culture medium

In order to confirm the injury degree of neonatal rat cardiomyocytes, the activities of LDH were measured with a Model 550 ELISA plate reader (Bio-Rad Laboratories Inc., Hercules, CA) according to the manufacturer’s instructions. The data in different groups were expressed as percentage.

### Cardiomyocyte apoptosis

Apoptosis was assessed using TUNEL assay in accordance with the manufacturer’s instructions. Cells were incubated in 0.1% Triton X-100 for 15 min on ice and covered with 80 μL TUNEL reaction mixture. All cell samples were incubated in a humidified chamber for 1 h at 37 °C in the dark and then stained with TUNEL (20 μg/mL). Finally, apoptotic cells were visualized in 10 selected fields and photographed at high-power magnification (×200) using an inverted fluorescence microscope (Olympus, Tokyo, Japan).

### Western blotting

Cytosolic and nuclear proteins were extracted from heart tissue and neonatal rat cardiomyocytes using NE-PER (Thermo Fisher Scientific, Waltham, MA) nuclear and cytoplasmic extraction reagents according to the manufacturer’s instructions. The protein concentration of each sample was measured using a Bio-Rad Protein Assay Kit (Bio-Rad Laboratories Inc., Hercules, CA). Proteins were separated using 10% sodium dodecyl sulphate-polyacrylamide gel electrophoresis (SDS-PAGE), and then transferred onto polyvinylidene difluoride (PVDF) membranes. Membranes were blocked with 5% non-fat dried milk at room temperature for 2 h, and then incubated with primary antibodies (NF-κB, p-IκB-α, ICAM-1, PCNA or β-actin) at 4 °C overnight. Membranes were incubated with HRP-conjugated secondary antibody for 1 h at room temperature. Immunolabelled proteins were detected using ECL-Plus reagent and blots were analyzed using Quantity One software (Bio-Rad Laboratories Inc., Hercules, CA).

### Statistical analysis

Data were presented as mean ± S.D. and analysed by GraphPad Prism software (La Jolla, CA). One-way ANOVA followed by Tukey’s test was applied for the statistical analysis of the parameters. Value of at *p* < 0.05 was considered statistically significant.

## Results

### Effects of APG on myocardial infarct size

**Figure 2.**
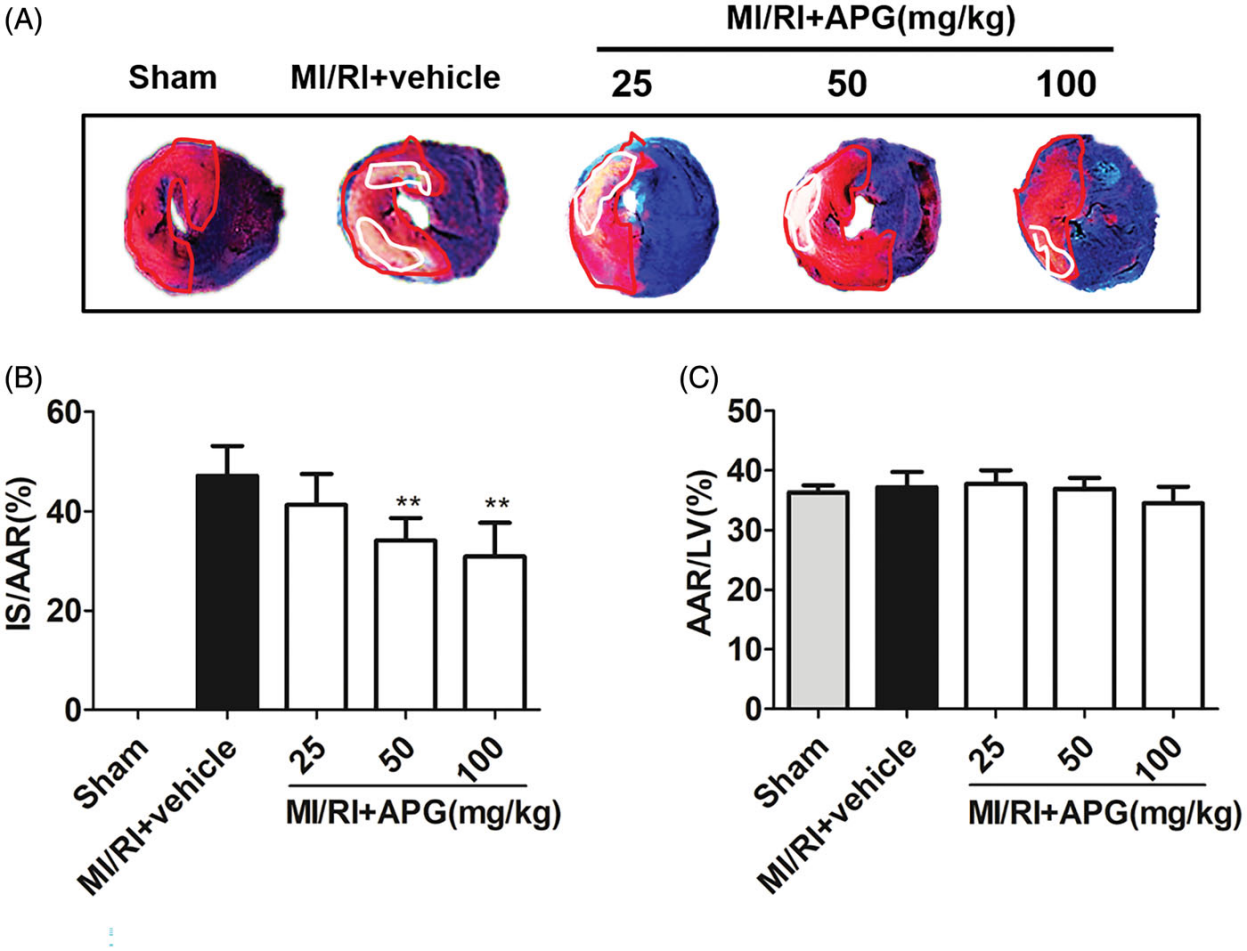
Area at risk and infarct size in different groups. (A) The representative photomicrographs of heart sections stained with Evans blue and TTC for different treatment groups are shown above the bar graph. (B) Treatment with APG (50 and 100 mg/kg) markedly reduced the infarct size caused by MI/RI. (C) AAR/LV was similar among groups. Data are expressed as mean ± S.D. (*n* = 6). Significance was determined by ANOVA followed by Tukey’s test. **p < 0.01 vs. MI/RI + vehicle. AAR: area at risk; LV: left ventricle; IS: infarct size.

No significant difference was observed in AAR/LV% between the vehicle and APG-treated groups, indicating that a comparable degree of ischaemic jeopardy existed between the groups after occlusion of the LAD (Figure 2(C)). However, treatment with 50 or 100 mg/kg APG significantly reduced the IS/AAR% compared with the MI/RI + vehicle group (Figure 2(A,B)).

### Effects of APG on cardiac histopathology

**Figure 3.**
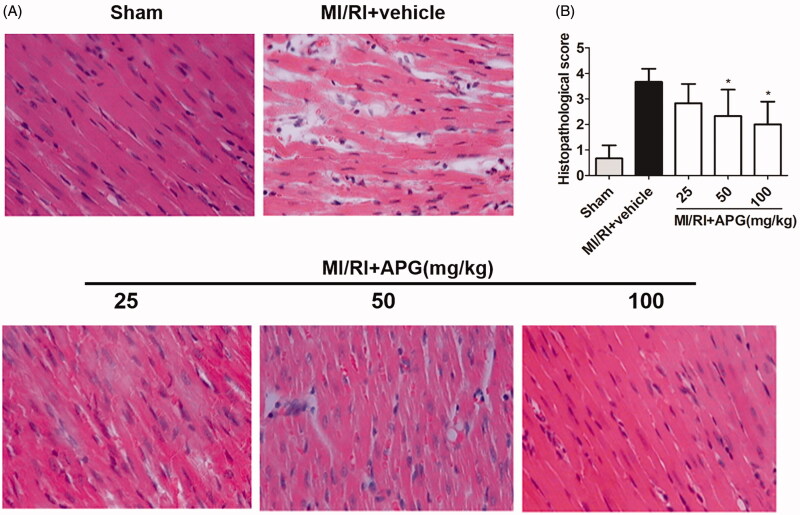
Representative light microscopic images of rat myocardial histopathological morphology (H&E, ×400). (A) Sham-operated group, the myocardial cell membrane showed clear integrity and no inflammatory cell infiltration. MI/RI + vehicle group, the myocardial tissue showed widespread cell structure disorder, large amount of infiltrated neutrophil granulocytes, interstitial oedema and myofibrillar fracture. MI/RI + APG (25 mg/kg) group, rats treated with APG (25 mg/kg) showed similar injury extended as vehicle group. MI/RI + APG (50 mg/kg) group, rats treated with APG (50 mg/kg) showed mild degree of necrosis and less infiltration of inflammatory cells. MI/RI + APG (100 mg/kg) group, rats treated with APG (100 mg/kg) showed nearly normal myofibrillar structure with few neutrophil granulocytes’ infiltration. (B) Effects of APG on myocardial histopathological scores. Values are expressed as the mean ± S.D. (*n* = 6). Significance was determined by ANOVA followed by Tukey’s test. *p < 0.05 vs. MI/RI + vehicle group.

In contrast to the sham-operated group, the MI/RI + vehicle group exhibited widespread myocardial structural damage, diffuse cloudy swelling, red-blood-cell extravasation, as well as infiltration of inflammatory cells. After that, APG (50 and 100 mg/kg) treatment groups ameliorated these myocardial injuries. It was revealed that morphological scores were notably lower in the APG-treated groups compared with vehicle group (Figure 3).

### Effects of APG on serum levels of CK-MB, LDH, TNF-α and IL-6

In this *in vivo* study, the serum levels of myocardial injury biochemical markers, CK-MB and LDH, were both noticeably higher in the vehicle group than those in the sham-operated group. In addition, treatment with APG markedly reduced the levels of both markers compared with the vehicle group. Significantly higher serum levels of TNF-α and IL-6 were also observed in the vehicle group than those in the sham-operated group, indicating the MI/RI-induced inflammatory response (Table 1). However, the release of these cytokines was greatly inhibited at the presence of APG (50 and 100 mg/kg) compared with the vehicle group.

**Table 1.** Effect of APG on levels of CK-MB, LDH, TNF-α, IL-6 and MPO for each group.

Groups	Dose (mg/kg)	CK-MB (ng/mL)	LDH (U/L)	TNF-α (pg/mL)	IL-6 (pg/mL)	MPO (U/mg)
Sham	0	33.2 ± 7.1	585.6 ± 114.7	42.6 ± 9.5	199.4 ± 33.6	1.53 ± 0.20
MI/RI + vehicle	0	76.9 ± 8.4^#^	1668.8 ± 257.9^#^	90.8 ± 15.3^#^	436.7 ± 20.6^#^	9.88 ± 1.79^#^
MI/RI + APG	25	69.0 ± 10.9	1573.7 ± 269.7	85.8 ± 13.6	411.8 ± 21.8	7.85 ± 1.95
	50	59.0 ± 10.6*	1251.2 ± 212.2*	75.0 ± 9.8*	297.1 ± 33.7*	7.80 ± 1.24
	100	50.7 ± 7.5*	980.8 ± 144.1*	59.3 ± 14.7*	272.9 ± 26.0*	7.13 ± 1.63*

Data are expressed as mean ± S.D. (*n* = 6), Significance was determined by ANOVA followed by Tukey’s test.

* *p* < 0.05 vs. MI/RI + vehicle.

# *p* < 0.05 vs. sham.

### Effects of APG on MPO activity in tissue

It was noted that MPO activity was very low in the sham-operated group, while markedly increased in the model group. Additionally, APG attenuated MPO activity in a dose-dependent manner (Table 1).

### Effects of APG on cell viability

**Figure 4.**
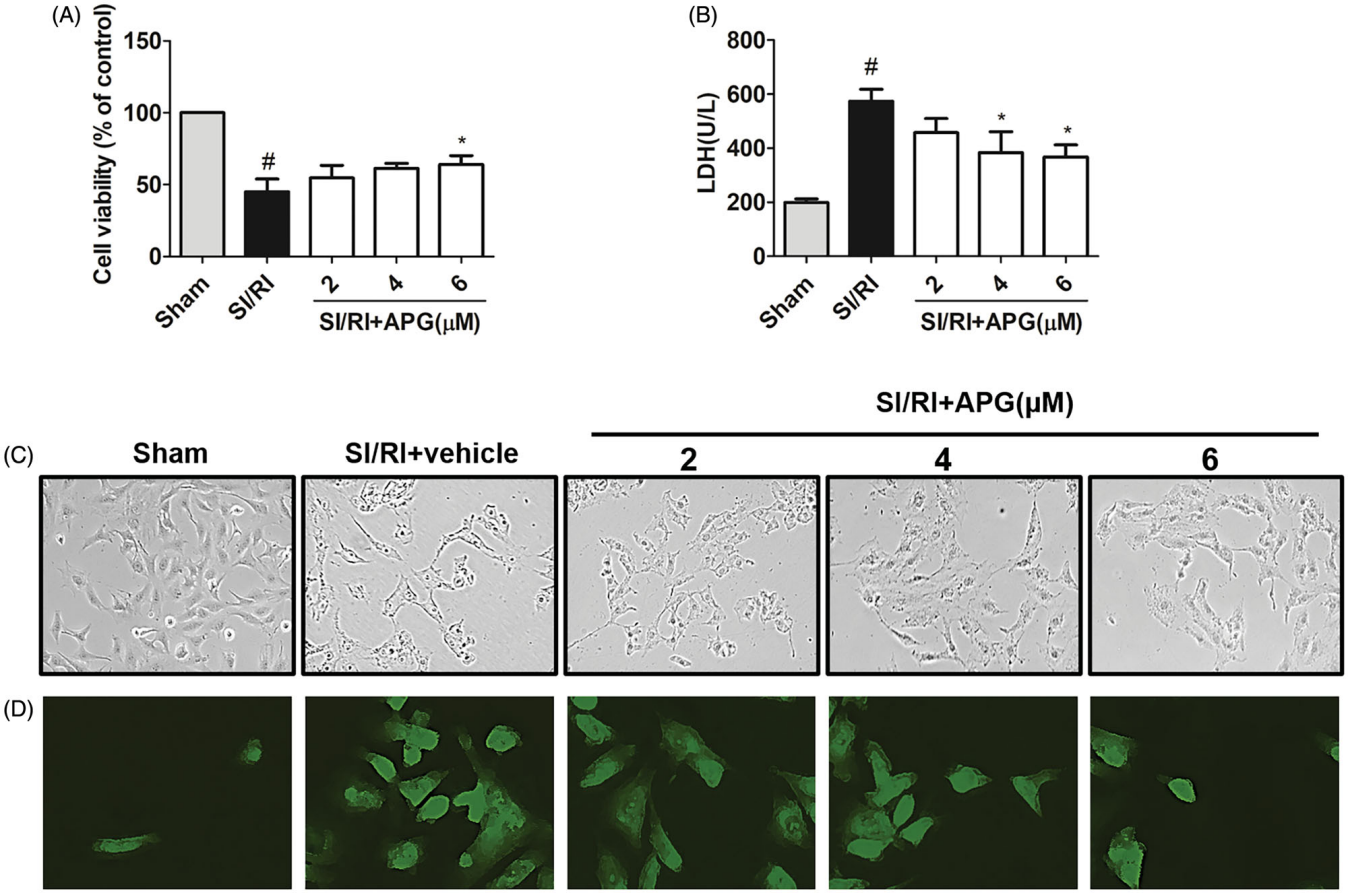
Effects of APG on cell viability, LDH release, cell morphology and apoptosis in neonatal rat cardiomyocytes subjected to SI/RI-induced injury. (A) Effects of APG on the viability of cardiomyocytes subjected to SI/RI-induced injury, measured by MTT assay. (B) Effects of APG on the release of LDH in the culture medium, measured by an ELISA kit. (C) Cells were subjected to SI/RI with or without different concentrations of APG. Cells were observed under an inverted phase contrast microscope. Treatment with APG resulted in less shrinkage compared with the SI/RI group. (D) Representative images of TUNEL staining were obtained. Significance was determined by ANOVA followed by Tukey’s test. Values represent as mean ± S.D. (*n* = 3). ^#^*p* < 0.05 vs. control group; *p < 0.05 vs. SI/RI group.

The results of cell viability are illustrated in Figure 4(A). It was revealed that APG at concentration of 6 µM significantly influenced cell viability.

### Effects of APG on LDH releases in culture medium

The release of LDH was used as an index of cardiomyocytes necrosis. It was found that LDH release was higher in the SI/RI group than that in control group, and a significant decrease of LDH activities was observed in the APG-treated groups relative to the SI/RI group (Figure 4(B)).

### Effects of APG on cell apoptosis

In control group, the majority of cardiomyocytes were viable. In addition, SI/RI stimulation induced apoptotic damage in cardiomyocytes compared with control group. However, in APG-treated groups, the apoptotic cardiomyocytes were markedly decreased compared with SI/RI group (Figure 4(C,D)).

### Intracellular mechanisms of APG

**Figure 5.**
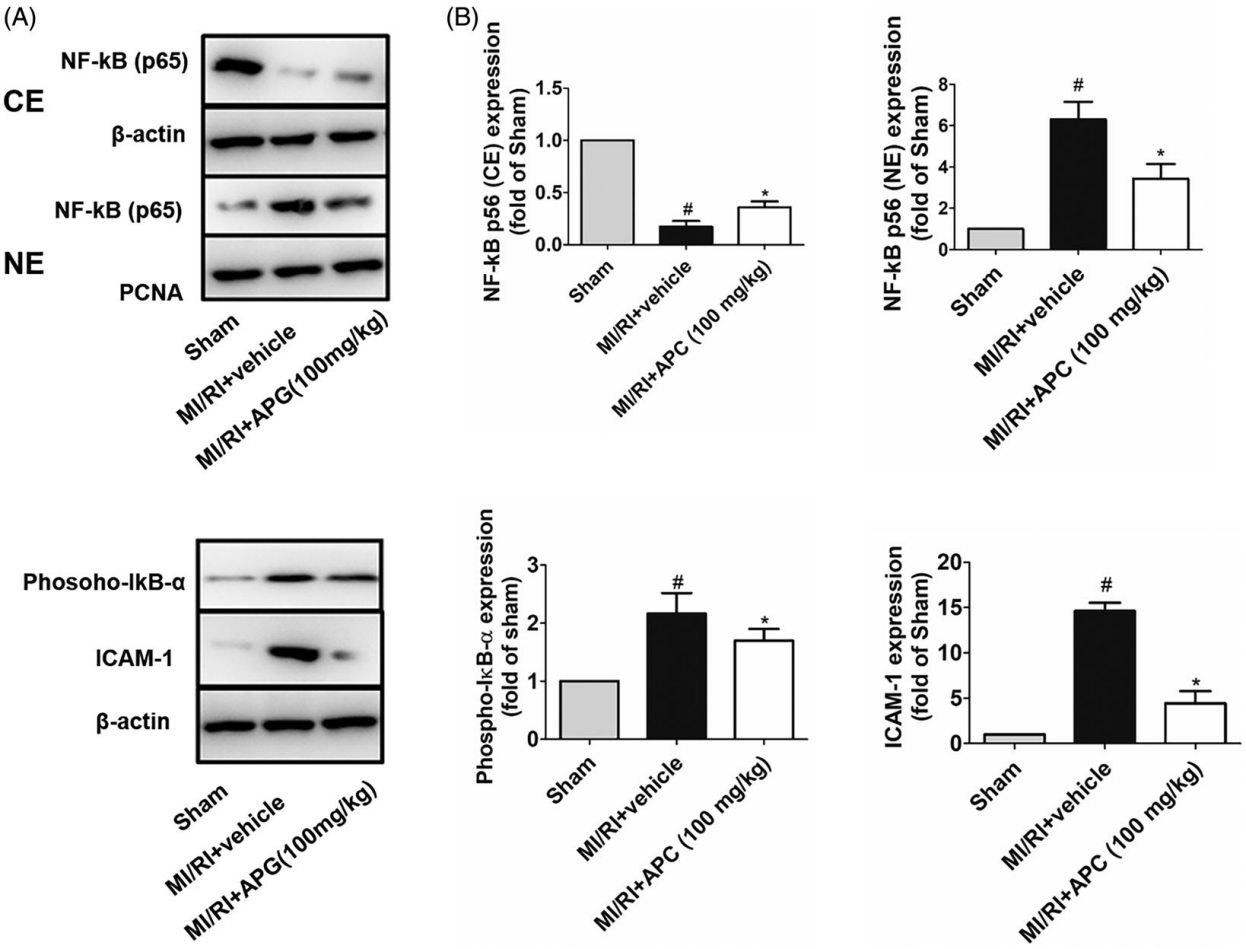
Effects of APG on translocation of NF-κB p65 and expression levels of phospho-IκB-α and ICAM-1 in MI/RI rats. (A) Representative blots of NF-κB p65 expression in cytosolic and nuclear fractions, and expression levels of phospho-IκB-α and ICAM-1 in rat myocardium. Nuclear extracts (NE) of NF-κB were normalized to PCNA (nuclear marker). Cytosolic extracts (CE) of NF-κB, phospho-IκB-α and ICAM-1 were normalized to β-actin (cytoplasmic marker). (B) Expression levels of NF-κB (NE), NF-κB (CE), phospho-IκB-α and ICAM-1 are shown as fold-change in sham-operated group. Significance was determined by ANOVA followed by Tukey’s test. Data are presented as mean ± S.D. (*n* = 3). ^#^*p* < 0.05 vs. Sham group, *p < 0.05 vs. MI/RI + vehicle group.

**Figure 6.**
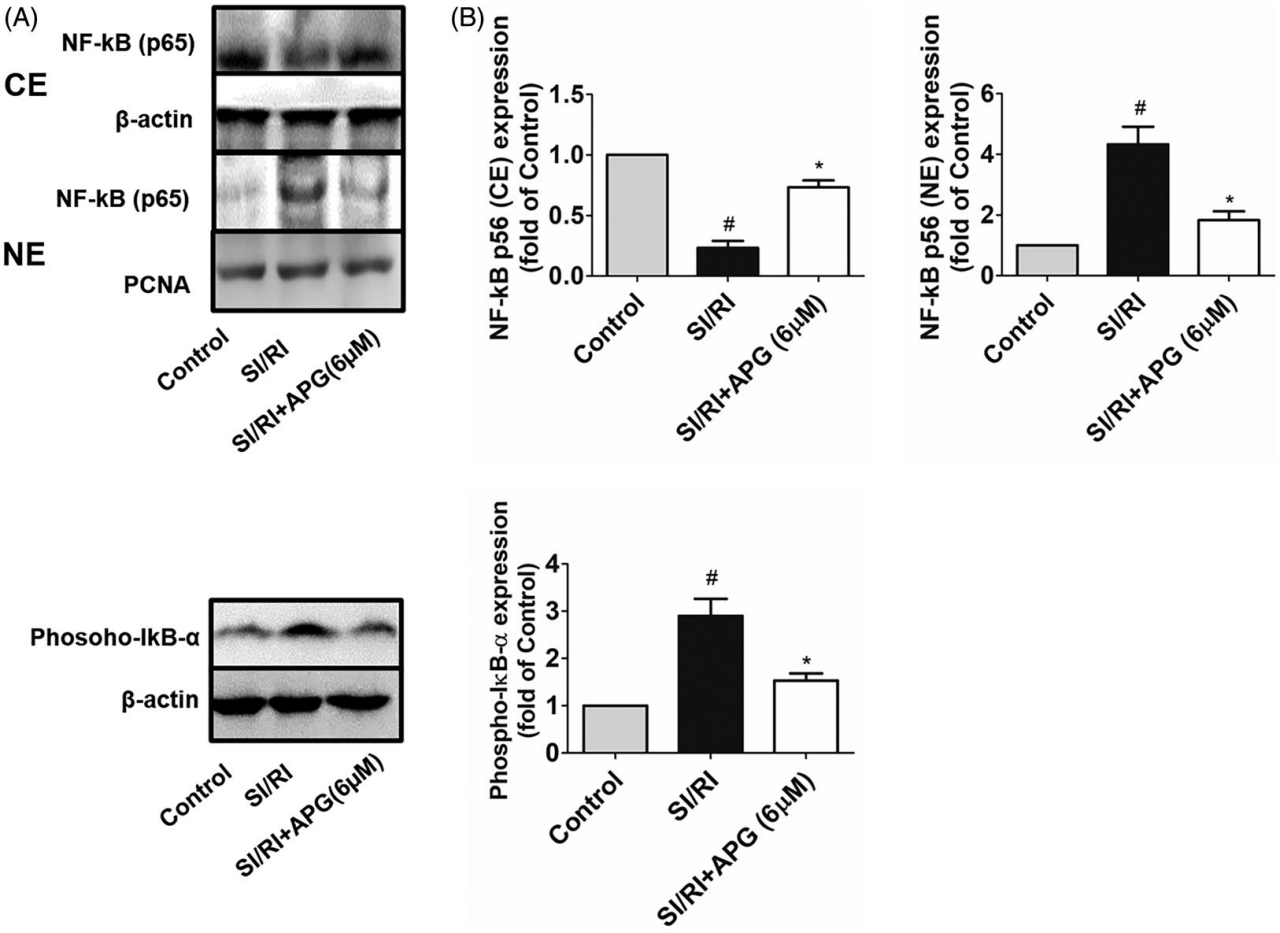
Effects of APG on NF-κB p65 translocation and expression level of phospho-IκB-α in SI/RI-stimulated rat cardiomyocytes. (A) Representative western blots of NF-κB p65 expression in cytosolic and nuclear fractions, and expression level of phospho-IκB-α in neonatal rat cardiomyocytes. Nuclear extracts (NE) of NF-κB were normalized to PCNA (nuclear marker). Cytosolic extracts (CE) of NF-κB, phospho-IκB-α and ICAM-1 were normalized to β-actin (cytoplasmic marker). (B) Expression levels NF-κB (NE), NF-κB (CE) and phospho-IκB-α are shown as fold change in the control group. Significance was determined by ANOVA followed by Tukey’s test. Data are presented as mean ± S.D. (*n* = 3). ^#^*p* < 0.05 vs. control group, *p < 0.05 vs. SI/RI group.

We investigated the effects of APG on NF-κB pathway by western blot analysis both *in vivo* and *in vitro*. In *in vivo* study, we found that the expression of NF-κB was increased in the nuclear fraction, whereas that was decreased in the cytoplasm after MI/RI, and the phospho-IκB-α expression was also enhanced in the vehicle group. However, the translocation of NF-κB into the nuclear fraction from the cytoplasm was suppressed and the phospho-IκB-α was significantly lower after APG administration (100 mg/kg). Additionally, MI/RI increased expression level of ICAM-1, which is a crucial adhesion molecule involved in inflammatory cascades induced by MI/RI. Notably, treatment with APG inhibited the expression level of ICAM-1 (Figure 5(A,B)). In the *in vitro* study, the expression level of NF-κB and phosphorylation of IκB-α in OGD/R-induced neonatal rat cardiomyocytes were investigated. In the SI/RI group, the cytosolic expression of NF-κB was significantly attenuated, while notably enhanced in the nuclear fraction. The phospho-IκB-α in neonatal rat cardiomyocytes significantly increased after OGD/R stimulation compared with control group. The results of the *in vitro* study showed an obvious similarity to the *in vivo* study, which suggested that APG may inhibit the NF-κB signalling pathway (Figure 6(A,B)).

**Figure 7.**
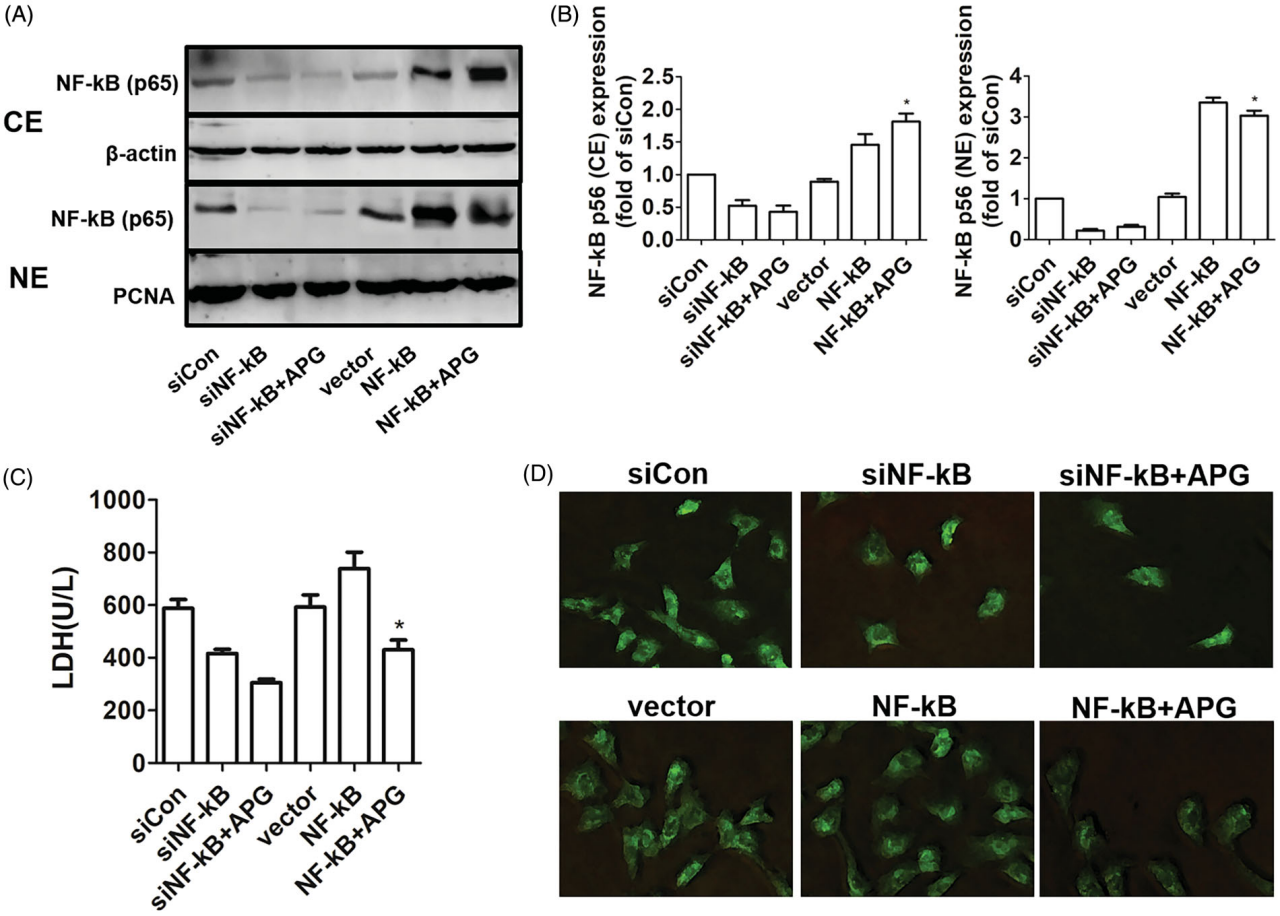
Effects of suppression or overexpression of NF-κB on the protective role of APG. (A) Representative western blots showed the expression levels of NF-κB (CE) and NF-κB (NE) when the cells were transfected with siNF-κB and pcDNA3.1(+)-NF-κB. (B) Expression levels of NF-κB (NE) and NF-κB (CE) are shown as fold change in the control group. (C) The release of LDH in the culture medium was measured by ELISA method when the cells were transfected. (D) Representative images of TUNEL staining were obtained when the cells were transfected. Significance was determined by ANOVA followed by Tukey’s test. Data are presented as mean ± S.D. (*n* = 3). *p < 0.05 vs. NF-κB group.

To further verify that the NF-κB signalling pathway could be involved in the protection of APG, the expression levels of NF-κB were inhibited and enhanced by using siRNA and pcDNA3.1(+) vectors, respectively. As displayed in Figure 7(A,B), we found that APG could noticeably attenuate the nuclear translation, involving overexpression of NF-κB after OGD/R stimulation. The result of LDH release and apoptotic damage confirmed that NF-κB signalling pathway could be implicated with the myocardial protection of APG. We also found that APG could decrease the LDH levels and apoptotic damage with overexpression of NF-κB after OGD/R stimulation (Figure 7(C,D)).

## Discussion

The present study demonstrated that APG, a flavonoid glycoside isolated from *Clematis tangutica*, has cardioprotective effects on rat model of MI/RI and OGD/R-induced injury in cultured neonatal rat cardiomyocytes. Our study provided evidence that treatment with APG significantly reduced the IS, decreased the serum levels of LDH and CK-MB in rat MI/RI model, and the LDH activity in OGD/R-induced cell culture medium. In addition, treatment with APG ameliorated MI/RI-induced histopathological lesions. The present study also revealed that APG reduced the inflammatory mediators. It also was noted that NF-κB was closely associated with the heart protective effects of APG. This conclusion was confirmed by increased NF-κB nuclear translocation and promoted phosphorylation of IκB-α after treatment with APG in both MI/RI rats and OGD/R-stimulated neonatal rat cardiomyocytes, indicating that APG plays a substantial role in protecting the heart from MI/RI through NF-κB signalling pathway.

At present, it is generally accepted that inflammation plays a critical role in MI/RI. In the early stages of MI/RI, inflammation promotes the local myocardial healing process. However, unrestrained inflammation in the infarcted heart has a direct deleterious effect on myocytes, increasing myocardial necrosis, thereby altering the long-term prognosis (Christia and Frangogiannis 2013). Timely suppression of the post infraction inflammatory reaction and rationally inflammation-modulating strategies might be beneficial for patients in clinical practice (Frangogiannis 2014). To our knowledge, TNF-α, IL-6 and ICAM-1 are essential inflammatory mediators involved in inflammatory response during MI/RI. Production and release of TNF-α is one of the initial events in the inflammatory response, which can trigger the production of other proinflammatory cytokines and upregulate the expression of adhesion molecules (e.g., ICAM-1) (Bazzoni and Beutler 1996). Meanwhile, synthesis of IL-6 is involved in promoting the expression level of ICAM-1 (Kukielka et al. 1995; Gwechenberger et al. 1999). The formation of mass ICAM-1 advances neutrophil adhesion and leukocyte infiltration (Kukielka et al. 1993), following release of large amounts of oxygen radicals, reactive oxygen species and cytotoxic molecules, resulting in cardiac dysfunction and myocardial cell death (Yucel et al. 2011). The present study confirmed that MI/RI increases serum levels of both TNF-α and IL-6. The results of western blotting indicated that MI/RI increases expression level of ICAM-1 in rat myocardial tissue. Notably, treatment with APG inhibited the increases of TNF-α and IL-6 in serum, and down-regulated the expression level of ICAM-1 in cardiac myocytes. The results indicated that APG ameliorates acute inflammatory injuries caused by MI/RI. This notion was also supported by the alteration of MPO activity. It has been reported that MPO accounts for 5% of the dry weight of the neutrophils, thus, the number of neutrophils in the myocardium could be estimated by MPO activity, and MPO could be therefore taken as an indicator of neutrophilic infiltration into consideration (Quan et al. 2013). In the current research, we found that MPO activity was enhanced in the cardiac tissue in the MI/RI group, while that was decreased in the APG-treated groups. The change of MPO activity indicated that APG could reduce the number of neutrophils and attenuate inflammation, which was consistent with pro-inflammatory cytokine results.

NF-κB proteins are a small family of related transcription factors, including Rel (cRel), RelA (p65), RelB, NF-κB1 (p50) and NF-κB2 (p52). They play significant roles in mounting immune responses and controlling expression levels of proinflammatory cytokines (Wan and Lenardo 2010). The main inducible form consisted of p65 and p50 subunits. As the earliest initiation factor of inflammation, NF-κB plays a pivotal role in MI/RI (Moss et al. 2007). It is activated rapidly in response to ischaemia in myocardium and this activation is further augmented by reperfusion (Hall et al. 2006). In its inactive form, the NF-κB dimer is localized to the cytoplasm and bound to an inhibitory protein known as IκB-α. MI/RI stimulates phosphorylation of IκB-α and its subsequent degradation via proteasomes. Activated NF-κB may translocate to the nucleus and up-regulate NF-κB-dependent inflammatory gene expression, leading to high expression of numerous inflammatory mediators, such as interleukins, cytokines and cell adhesion molecules (Valen et al. 2001). In the present study, we used OGD/R induced the neonatal rat cardiomyocytes and found the inhibitory effect of APG on NF-κB pathway. The results demonstrated that APG decreased expression level of phospho-IκB-α and inhibited the translocation of NF-κB both *in vivo* and *in vitro*. The current research suggested that the protective effects of APG may be largely due to suppression of the inflammatory response via inhibition of NF-κB activation. To further confirm that NF-κB could be involved in the myocardial protection of APG, the expression level of NF-κB was also regulated by transfection with siRNA-NF-κB and pcDNA3.1(+)-NF-κB *in vitro.* Our findings revealed that the protection of APG was negatively correlated with the translocation of NF-κB. These results further demonstrated that could APG mediate translocation of NF-κB, resulting in myocardial protection against MI/RI.

## Conclusions

The present study presented evidence that APG contains cardioprotection effects both *in vivo* and *in vitro*. Our findings showed that treatment with APG reduced myocardial IS, ameliorated histopathology lesions and inhibited the releases of CK-MB and LDH in MI/RI rat model. In addition, pre-treatment with APG saved cell viability and decreased the leakage of LDH in OGD/R-induced neonatal rat cardiomyocytes. It also was found that these protective effects of APG were associated with decreased levels of TNF-α and IL-6, as well as the content of MPO and the expression of ICAM-1. Furthermore, both *in vivo* and *in vitro* studies proved that APG could inhibit phospho-IκB-α levels and prevent the nuclear translocation of NF-κB. Thus, it is likely that APG protects the heart mainly through suppression of the inflammatory response via inhibition of NF-κB activation.
